# Effects of different fractions of inspired oxygen on delayed neurocognitive recovery in patients with one-lung ventilation: a randomized clinical trial

**DOI:** 10.4103/mgr.MEDGASRES-D-25-00060

**Published:** 2026-01-06

**Authors:** Chujun Wu, Wangzhi Zhang, Jun Lin, Dizhou Zhao, Jieyu Fang

**Affiliations:** 1 Department of Anesthesiology, The First Affiliated Hospital, Sun Yat-sen University, Guangzhou, Guangdong Province, China; 2 Department of Anesthesiology, Sun Yat-sen Memorial Hospital, Sun Yat-Sen University, Guangzhou, Guangdong Province, China; 3 Department of Anesthesiology, Guangzhou Women and Children’s Medical Center, Guangzhou Medical University, Guangzhou, Guangdong Province, China; 4 Department of Anesthesiology, Shenzhen People’s Hospital, The Second Clinical Medical College, Jinan University, The First Affiliated Hospital, Southern University of Science and Technology, Shenzhen, Guangdong Province, China; 5 Department of Anesthesiology, Guangxi Hospital Division of The First Affiliated Hospital, Sun Yat-sen University, Nanning, Guangxi Zhuang Autonomous Region, China

**Keywords:** anesthesia, blood analysis, cerebral oxygen saturation, cognitive function, delayed neurocognitive recovery, fractions of inspired oxygen, inflammatory response, lung surgery, one-lung ventilation, oxidative stress

## Abstract

Delayed neurocognitive recovery is an important cause of morbidity and disability after surgery. This perspective randomized clinical study mainly explored the effects of different fractions of inspired oxygen (FiO_2_) on Delayed neurocognitive recovery in patients with one-lung ventilation. Between December 2021 and July 2022, 88 patients aged 30–70 years who underwent thoracoscopic pulmonary lobectomy with one-lung ventilation at the First Affiliated Hospital of Sun Yat-sen University, China, were divided into two groups. One group received 70% FiO_2_ (70% FiO_2_ group), while the other received 100% FiO_2_ (100% FiO_2_ group) during one-lung ventilation. The outcomes were the incidence of delayed neurocognitive recovery on postoperative days 1 and 3, the incidence of cerebral oxygen desaturation during surgery, and the perioperative level of blood gas, inflammatory response, and oxidative stress. The incidence rates of cerebral oxygen desaturation and delayed neurocognitive recovery on postoperative days 1 and 3 were similar between the two groups. Compared with the 70% FiO_2_ group, in the 100% FiO_2_ group, the oxygenation index was significantly higher at 30 minutes after one-lung ventilation and 60 minutes after one-lung ventilation, and the arterial partial pressure of carbon dioxide was significantly lower at 15, 30 and 60 minutes after one-lung ventilation, while that of superoxide dismutase was significantly lower at 15 minutes after one-lung ventilation. Compared with 70% FiO_2_, 100% FiO_2_ did not increase the incidence of delayed neurocognitive recovery on days 1 and 3 post-operatively in patients with one-lung ventilation.

## Graphical abstract

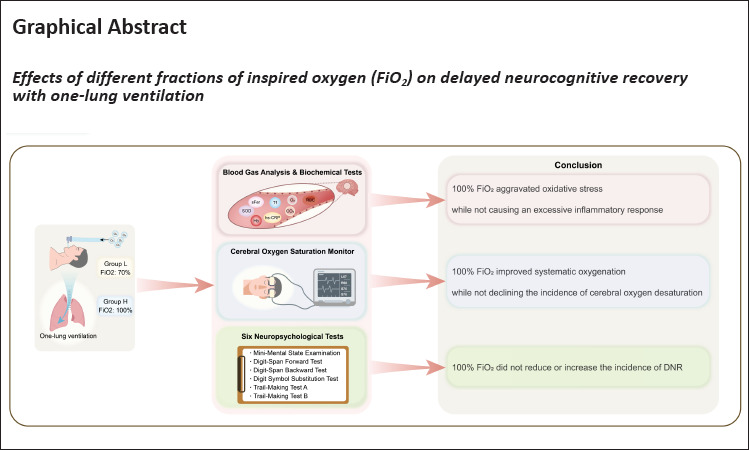

## Introduction

Postoperative cognitive impairment is an important cause of morbidity, disability, and death after surgery.^1^ In addition, it has a socio-economic impact, as it forces patients out of the labor market and makes patients need more special care in terms of social transfer.^2^,^3^ It has been previously reported that 17–56% of patients develop cognitive dysfunction after surgical procedures.^4^ In 2018, cognitive impairment or a change in the interval from 0 to 30 days postoperatively was defined as delayed neurocognitive recovery (DNR), previously known as postoperative cognitive dysfunction.^5^ DNR mainly manifests as damage to memory, cognitive ability, language ability, or other aspects of cerebral function.^6^

Although several mechanisms have been proposed to be involved in the development of DNR, its cause is still unknown. Many clinical and animal studies have suggested that the inflammatory process plays a significant role in cognitive dysfunction.^7^,^8^,^9^,^10^ The key feature of neuroinflammation is the activation of microglia. Produced and secreted by activated microglia, proinflammatory factors, such as some active oxides, mediate tissue injury, inflammation, apoptosis, autophagy, necrosis, vessel dilation, neurodegeneration, neuronal apoptosis, and neuro-dysfunction.^10^,^11^

One-lung ventilation (OLV) is a technique for supplying artificial respiration to a single lung in thoracic surgical procedures. This non-physiological ventilation creates an imbalance in the ratio of ventilation and blood flow, which increases the risk of cerebral hypoxemia.^12^ In some studies, the balance between cerebral oxygen delivery and consumption was assumed to be associated with the development of DNR.^13^,^14^ However, unanimous agreements have not been reached regarding the relationship between cerebral oxygen balance and DNR.^15^

Actually, to avoid myocardial and cerebral hypoxia, patients who undergo OLV are conventionally treated with pure oxygen ventilation.^16^ However, this measure is controversial in the international anesthesiology community.^17^ Many researchers supposed that the use of a high concentration of inspired oxygen would lead to hyperoxia, which is potentially harmful.^18^ Furthermore, toxicity arising from high oxygen is often thought to be mediated through ‘oxidative stress.’^17^,^19^,^20^,^21^ The brain is extremely vulnerable to oxidative stress because the content of antioxidants in it is relatively low. Therefore, oxidative stress is considered an important risk factor for DNR.^22^

Although current research on the pathogenesis of DNR is mostly focused on neuroinflammation, the specific etiology and pathophysiology of DNR are not clear.^15^ Whether high inhaled oxygen concentrations increase the risk of DNR has not been determined in anesthesiology.

Currently, anesthesiologists are becoming more aware of the roles that oxidative stress or impaired cerebral oxygen balance may play in neuroinflammation and subsequent DNR. However, there are few studies on how intraoperative fractions of inspired oxygen (FiO_2_) affect this.^17^

This randomized controlled trial was designed to assess the effects of different intraoperative FiO_2_ values in patients undergoing thoracic surgery on the incidence of DNR on postoperative days 1 and 3. Moreover, we investigated whether the change in FiO_2_ impacts the cerebral oxygen balance during OLV and the perioperative level of inflammatory response and oxidative stress.

## Methods

### Design

This study, conducted between December 2021 and July 2022, was designed as a single-center, double-blinded, randomized controlled trial. The study was approved by the Clinical Research Ethics Committee of the First Affiliated Hospital of Sun Yat-sen University, China (approval No. 2020-423; October 22, 2020), and was registered with the Chinese Clinical Trial Registry (ChiCTR2100045495) before patient enrollment. All participants signed informed consent before surgery and agreed to be included in the study. The trial was designed to assess the effect of two intraoperative inhaled oxygen fractions (0.7 and 1.0) on DNR incidence among patients undergoing thoracic surgery.^18^ All experimental procedures were performed in strict accordance with the Declaration of Helsinki, and the trial was performed according to the CONsolidated Standards Of Reporting Trials (CONSORT) guidelines.^23^

### Participants

Participants aged 30–70 years, with an American Society of Anesthesiologists physical status^24^ of 1 or 2 and a preoperative Mini-Mental State Examination (MMSE) score^25^ > 23, who were scheduled for elective pulmonary lobectomy with OLV of an expected duration > 60 minutes under thoracoscopy were enrolled in the study. The exclusion criteria included: (1) low hemoglobin (Hb < 100 g/L) and hypoproteinemia; (2) a history of dementia or psychiatric illness; (3) current use of sedatives, antidepressants, or corticosteroids; (4) alcoholism and drug dependence; (5) difficulty with follow-up or poor compliance; and (6) aphasia, hearing or visual impairment, or any communication impediment.

At the same time, we recruited 34 non-surgery control subjects. They were community volunteers who were age-matched to the surgical patients. These non-surgical control subjects were specifically recruited to adjust for the practice effect of repeated neuropsychological tests used in this study, using the same inclusion and exclusion criteria as those used for the surgical patients.

### Randomization and blinding

A study statistician who was not involved in this trial generated random numbers without restriction (simple randomization). The randomization sequences were sealed in sequentially numbered, opaque envelopes and sequentially sent to an anesthesiologist when the patients were entering the operating room. The anesthesiologist opened the numbered envelope to obtain and implement the allocated intervention regimen but did not know any experimental data during the surgery. Independent research assistants tested blood samples, monitored cerebral oxygenation and evaluated cognitive function, were not involved in perioperative patient care and were blinded to patient allocation, as were patients and surgeons.

### Anesthetic management and perioperative care

Anesthesia was standardized in this trial. All patients fasted for 8 hours before surgery. Intraoperative standard monitoring included electrocardiography, blood pressure, heart rate, pulse oxygen saturation, end-tidal carbon dioxide partial pressure, core body temperature, Narcotrend (Monitor Technik, Bad Bramstedt, Germany), and cerebral regional oxygen saturation (rSO_2_) (INVOS 5100C, Somanetics Covidien, Minneapolis, MN, USA).

Anesthesia was induced with propofol (2 mg/kg), sufentanil (0.8 μg/kg), and cis-atracurium (0.2 mg/kg). Anesthesia was maintained with a target-controlled infusion of propofol (2.0–3.0 μg/mL) and remifentanil (4.0–6.0 ng/mL) to keep the heart rate and blood pressure within the basal value of 20%. Air warming was used to maintain the core body temperature near 36.0 °C.

Radial artery cannulation was performed to obtain arterial blood samples. To obtain jugular bulb blood samples, a 16G single-cavity central venous catheter was placed retrograde, as opposed to central venous catheterization, via the right internal jugular vein to a distance equal to that measured from the point of insertion to the level of the right mastoid process (approximately the level of the right jugular bulb) or until resistance was met, indicating that the tip of the catheter had reached the jugular bulb.^26^ The blood samples were analyzed using a blood gas analyzer (GEM® Premier™ 3000 blood gas analyzer, Bedford, MA, USA).

After manual ventilation via a face mask with 100% oxygen for 3 minutes, all patients in this study were intubated with a double-lumen tube, and the correct position was confirmed using a flexible bronchoscope. The mechanical ventilatory volume was set at 8 mL/kg (predicted body weight) for two-lung ventilation, and OLV was started at 6–8 mL/kg (predicted body weight) according to the recommended ventilation strategy for lung surgery.^27^ The ventilation rate was adjusted to maintain the end-tidal carbon dioxide partial pressure at 35–45 mmHg during surgery.

### FiO_2_ management

Patients received 70% or 100% oxygen after securing the airway. This strategy of an FiO_2_ was utilized throughout the intraoperative period.

### Data collection

#### Fundamental data collection

The preoperative evaluation was performed 1 day before surgery. Fitness and functionality before surgery were assessed with the American Society of Anesthesiologists physical status classification. The durations of OLV, surgery, and anesthesia were recorded.

#### Cerebral oxygen saturation monitoring

We acquired a cerebral rSO_2_ value for patients in a quiet and steady state before anesthesia induction as a baseline while the patient was breathing room air in the operating room. The cerebral rSO_2_ values were also recorded at the following time points: 15 minutes before OLV (T_1_), 15 minutes after OLV (T_2_), 30 minutes after OLV (T_3_), 60 minutes after OLV (T_4_), and 15 minutes after termination of OLV (T_5_).

#### Blood gas analysis

Arterial and jugular bulb venous blood samples (2 mL) were obtained for blood gas analysis at T_1-5_. The following parameters were measured: arterial oxygen saturation (SaO_2_), jugular bulb venous oxygen saturation (SjO_2_), arterial partial pressure of carbon dioxide (PaCO_2_), jugular bulb venous partial pressure of carbon dioxide (PjCO_2_), oxygenation index (OI), difference in alveolar arterial oxygen partial pressure (PA-aO_2_), and Hb.

#### Definition of cerebral oxygen desaturation

Normal rSO_2_ values range between 58% and 82% (70 ± 6%).^28^ SjO_2_ is normally 55–75%.^26^ Cerebral oxygen desaturation, prognosticating the possible presence of cerebral hypoxia, is defined as (1) an absolute SjO_2_ < 50%, (2) an absolute rSO_2_ < 50%, or (3) a relative decrease in right, left, or average rSO_2_ > 20% compared with baseline for each patient at any measurement time point.^15^,^29^,^30^,^31^

#### Biochemical tests

Intravenous blood samples (5 mL) were collected from the basilic vein for the oxidative stress test 15 minutes before OLV (T_i_), 30 minutes after OLV (T_ii_), and postoperative day 1 (T_iii_). The measured biochemical parameters included transferrin (Tf), serum ferritin (sFer), ischemia-modified albumin (IMA), superoxide dismutase (SOD), and high-sensitivity C-reactive protein (hs-CRP).

#### Neuropsychological assessment

To assess cognitive function, a battery of six neuropsychological tests was conducted on day 1 pre-operatively (baseline) and days 1 and 3 post-operatively, including the (1) MMSE, (2) digit-span forward test, (3) digit-span backward test, (4) digit symbol substitution test, (5) trail-making test A, and (6) trail-making test B.^25^

All of these cognitive tests were performed in a quiet hospital room by an investigator who was blinded to the random allocation and not involved in the procedure of anesthetic management. These tests were also performed with the non-surgery control subjects in a quiet room at the same intervals as the surgical patients.

#### Diagnostic criteria for delayed neurocognitive recovery

To diagnose DNR, we used the definition from the International Study of Post-Operative Cognitive Dysfunction 1.^32^ For controls, test scores on days 1 and 3 were compared with scores on the day before surgery. For each test, we calculated the difference (*Xdc*) for each participant and the mean difference (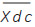) for all participants in the control group, and then the standard deviation of the differences (*SD*[*Xdc*]). 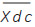 may be taken as the estimated average learning effects, taking into account the fact that people get better the second time they do a test, even if there is no change in whatever is being measured. For patients, scores on the five neuropsychological tests on post-operative days 1 and 3 were compared with scores on the same tests completed the day before surgery. Similarly, we obtained the difference (*Xd*) of each test for each individual patient.

The changes in test scores for an individual patient undergoing surgery were compared with the changes in the test scores of the control group over the same interval. We finally obtained a *Z* score using the following equation: *Z* = (*Xd* – 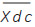)/*SD*(*Xdc*). Notably, for each individual patient, we could obtain not only a *Z* score of each test but also a combined *Z* score of all tests via another equation: *Z*_combined_ = (∑*Z*)/*SD*(∑*Zc*). ∑*Z* is a summation of the *Z* scores of all tests for an individual patient, and *SD*(∑*Zc*) was calculated on the basis of ∑*Zc* for all controls.^33^

DNR was defined as a decline in cognitive tests from baseline compared with controls, as expressed by an individual’s *Z* score on at least two individual tests or a *Z*_combined_ score ≥ 1.96. This definition considers general deterioration in all tests or significant deterioration in only some tests.^34^

### Statistical analysis

The DNR incidence on post-operative day 1 was the primary outcome of the study. The DNR incidence on post-operative day 3, cerebral desaturation incidence, hs-CRP level and oxidative stress index were the secondary outcomes. Data were expressed and tested based on their distribution (calculated by the Shapiro–Wilk test). Continuous variables are reported as the mean ± standard deviation (SD) or median (inter-quartile range) and were compared using the Student’s *t*-test or the Mann–Whitney *U* test. Categorical data are reported as frequencies (percentages) with 95% confidence intervals and were analyzed using chi-square test or Fisher’s exact test. Two-way repeated-measures analysis of variance followed by the Bonferroni post hoc test were used to assess the effect of the interaction term, within-group comparisons over time, and between-group comparisons over time. Differences were considered statistically significant at *P* < 0.05. All calculations and statistical analyses were performed using SPSS Statistics software (release 24.0; IBM Corp., Armonk, NY, USA).

### Sample size calculation

The sample size was calculated using previous trial data on DNR incidence, which was approximately 20.5% in comparable patient populations.^35^ Forty-three patients in each surgery group were required to provide 80% power for detecting a 20% difference in the primary outcome, with a two-tailed significance level at 0.05 and a 10% dropout rate. PASS 11.0 (NCSS, LLC, Kaysville, UT, USA) software was used to estimate the required sample size in this study.

## Results

**Figure 1:**
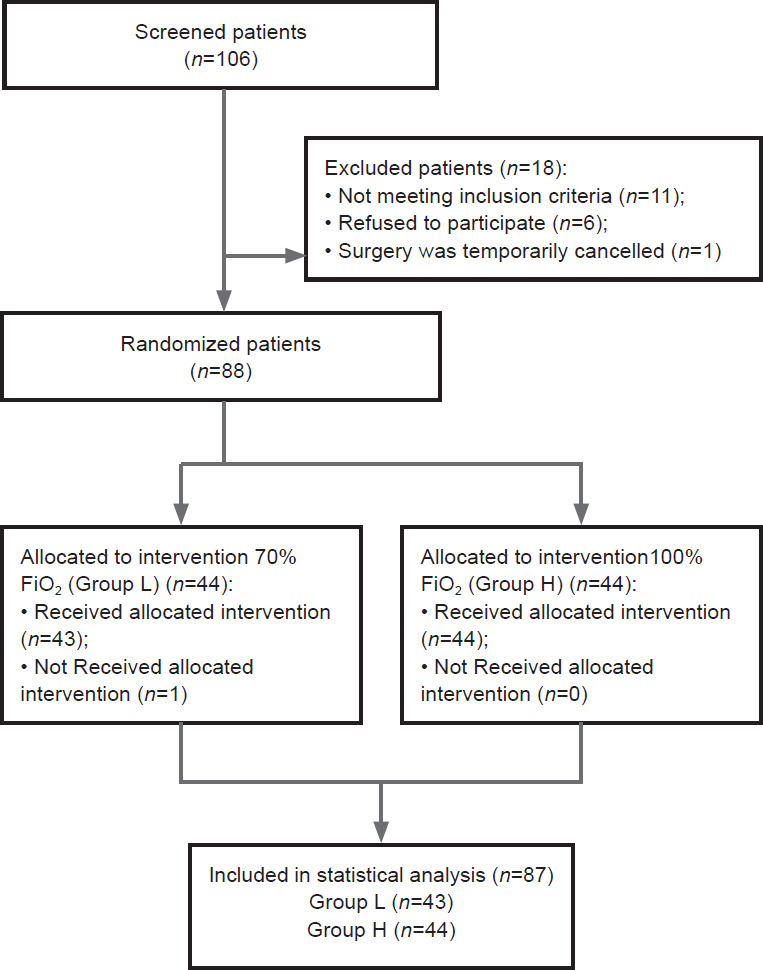
Flow chart of the trial inclusion process. FiO_2_: Fractions of inspired oxygen; Group L: 70% inspired oxygen group; Group H: 100% inspired oxygen group.

A total of 106 patients were screened for this study. Among them, 6 patients refused to participate, 11 patients did not meet the inclusion criteria, 1 patient was discharged on the day of the operation, and the operation was temporarily canceled. Thus, a total of 88 patients were enrolled, 44 of whom maintained FiO_2_ at 70% during OLV (70% FiO_2_ group) and 44 of whom maintained FiO_2_ at 100% during OLV (100% FiO_2_ group). One patient in the 70% FiO_2_ group was excluded because he needed pure oxygen combined with PEEP to maintain pulse oxygen saturation ≥ 92% during OLV. Finally, 87 patients completed the trial and were included in the statistical analysis (70% FiO_2_ group: *n* = 43; 100% FiO_2_ group: *n* = 44) (**Figure 1**).

Baseline participant characteristics, including the preoperative MMSE score, preoperative Hb, and basal rSO_2_ value, are shown in **Table 1**.

**Table 1:** Baseline demographic and clinical characteristics of patients with OLV with different FiO_2_ values and delayed neurocognitive recovery

	70% FiO_2_ group (*n*=43)	100% FiO_2_ group (*n*=44)
Age (yr)	56.19±9.68	52.80±10.63
Body mass index (kg/m^2^)	23.00±2.51	23.46±2.89
Sex		
Female	24 (57)	22 (50)
Male	19 (44)	22 (50)
Years of education	5.26±3.79	5.73±4.06
ASA physical status		
I	32 (74)	35 (80)
II	11 (26)	9 (20)
Comorbidity		
Hypertension	7 (16)	6 (14)
Diabetes mellitus	4 (9)	2 (5)
Coronary artery disease	0	1 (2)
Pulmonary infection	2 (5)	0
Operative position		
Left lateral decubitus	25 (58)	27 (61)
Right lateral decubitus	18 (42)	17 (39)
Duration of anesthesia (min)	117.14±30.66	109.27±44.59
Duration of surgery (min)	105.00 (90.00-126.50)	94.50 (75.00-111.25)
Duration of OLV (min)	90.00 (75.50-110.00)	79.00 (65.00-103.75)
Mini-mental state examination score	28.00 (26.00-29.00)	29.00 (27.00-29.00)
Preoperative hemoglobin (g/L)	133.44±15.82	137.48±16.74
Basal regional cerebral oxygen saturation (%)		
Left	71.27±8.10	74.55±7.77
Right	70.73±9.23	73.84±8.16

The data are presented as number (percentage), means ± SD or median (IQR). ASA: American Society of Anesthesiologists; FiO_2_: fractions of inspired oxygen; OLV: one-lung ventilation.

### Incidence of delayed neurocognitive recovery

There was no significant difference in the incidence of DNR between the two groups on post-operative days [7 of 43 (16%) patients *vs*. 2 of 44 (5%) patients; *P* = 0.09]. There was also no significant difference in the incidence of DNR between the two groups 3 days post-operatively [1 of 43 (2%) patients *vs*. 0 of 44 (0%) patients; *P* = 0.49; **Table 2**].

**Table 2:** Demographic and clinical data of all subjects

	70% FiO_2_ group (*n* = 43)	100% FiO_2_ group (*n* = 44)	*P*_Time_	*P*_Group_	*P*_Interaction_
SOD (U/mL)					
T_i_	176.92±18.12	169.81±17.40	< 0.001	0.41	0.005
T_ii_	165.59±24.27^#^	154.63±18.52^*#^			
T_iii_	185.81±19.44^#^^†^	194.60±28.95^#^^†^			
Tf (g/L)					
T_i_	1.93±0.29	2.06±0.32	< 0.001	0.21	0.25
T_ii_	1.86±0.34	2.00±0.33			
T_iii_	1.70±0.33	1.94±0.38			
sFER (μg/L)					
T_i_	227.34±139.12	227.11±192.19	< 0.001	0.95	0.41
T_ii_	218.35±130.48	222.27±186.72			
T_iii_	274.05±144.64	263.79±198.54			
IMA (U/mL)					
T_i_	81.32±3.21	82.63±2.77	< 0.001	0.49	0.12
T_ii_	83.04±3.57	83.57±3.01			
T_iii_	85.69±4.82	85.36±4.84			
Hs-CRP (mg/L)					
T_i_	1.71±2.40	1.40±2.00	< 0.001	0.84	0.61
T_ii_	3.34±10.11	1.52±2.63			
T_iii_	99.63±53.70	98.73±77.10			
OI					
T_1_	445.57±107.01	430.79±72.05	< 0.001	0.17	0.01
T_2_	197.87±93.74^§^	243.98±106.76^§^			
T_3_	176.24±76.61^§^	237.56±113.97^§^			
T_4_	196.65±93.25^§^	275.36±128.40^§^			
T_5_	409.96±83.27^≠^&^	379.61±113.92^≠^&^			
PA-aO_2_ (mmHg)					
T_1_	151.94±73.34	243.86±81.55	< 0.001	< 0.001	0.77
T_2_	307.65±69.62	450.21±89.71			
T_3_	332.06±71.21	447.10±102.34			
T_4_	321.53±90.68	413.46±109.50			
T_5_	179.84±78.68	280.78±85.93			
PaCO_2_ (mmHg)					
T_1_	40.06±6.20	41.45±5.40	0.56	0.07	0.007
T_2_	44.12±5.94^§^	39.59±4.83			
T_3_	43.53±5.85	38.97±4.80			
T_4_	44.31±6.50^§^	38.93±5.16			
T_5_	42.09±6.75	41.10±6.50			
PjCO2 (mmHg)					
T_1_	55.29±5.72	53.62±4.86	0.04	0.01	0.09
T_2_	54.90±5.13	52.17±4.98			
T_3_	55.58±5.60	52.38±4.58			
T_4_	55.68±6.15	51.52±4.75			
t_5_	56.07±5.97	55.10±4.93			
SaO_2_ (%)					
T_1_	99.93±0.35	100.00±0.00	<0.001	0.008	0.12
T_2_	97.56±2.65	99.32±1.56			
T_3_	97.51±2.12	99.03±1.92			
T_4_	98.09±1.77	99.32±1.96			
t_5_	99.91±0.53	99.96±0.21			
SjO_2_ (%)					
T_1_	65.42±14.48	65.42±12.41	0.005	> 0.99	0.41
T_2_	58.03±12.64	60.19±10.79			
T_3_	58.53±13.45	58.02±11.50			
T_4_	56.68±13.39	58.03±12.11			
t_5_	57.63±13.61	60.38±12.72			
Hb (g/L)					
T_1_	123.06±14.40	125.57±17.37	0.06	0.8	0.26
T_2_	126.47±14.24	116.76±26.02			
T_3_	121.81±15.06	118.38±16.35			
T_4_	124.00±19.20	119.46±16.13			
T_5_	119.88±12.63	121.78±16.75			
Delayed neurocognitive recovery					
Postoperative day 1	7 (16)	2 (5)	0.09		
Postoperative day 3	1 (2)	0	0.49		
Cerebral oxygen desaturation	16 (37)	17 (39)	0.97		
SjO_2_ desaturation	16 (37)	16 (36)	0.94		
rSO_2_ desaturation	2 (5)	2 (5)	0.98		

The data are presented as mean ± SD or number (percentage), and were analyzed by two-way repeated-measures analysis of variance or chi-square test. ^*^P = 0.025, *vs*. 70% FiO_2_ group; ^#^*P* < 0.05, *vs.* T_i_; ^†^*P* < 0.05, *vs.* T_ii_; ^§^*P* < 0.05, *vs.* T_1_^≠^*P* < 0.05, *vs.* T_2_; ^^^*P* < 0.05, *vs.* T_3_; ^&^*P* < 0.05, *vs.* T_4_. FiO_2_: Fractions of inspired oxygen; Hb: hemoglobin; hs-CRP: hypersensitive C-reactive protein; IMA: ischemia-modified albumin; OI: oxygenation index; OLV: one-lung ventilation; PA-aO_2_: alveolar arterial oxygen partial pressure difference; PaCO_2_: arterial partial pressure of carbon dioxide; PjCO_2_: jugular bulbar venous partial pressure of carbon dioxide; rSO_2_: cerebral regional oxygen saturation; SaO_2_: arterial oxygen saturation; sFER: serum ferritin; SjO_2_: jugular bulbar venous oxygen saturation; SOD: superoxide dismutase; T_1_:15 minutes before OLV; T_2_: 15 minutes after OLV; T_3_: 30 minutes after OLV; T_4_: 60 minutes after OLV; T_5_: 15 minutes after termination of OLV; T_i_: 15 minutes before one-lung ventilation; T_ii_: 30 minutes after one-lung ventilation; T_iii_: 1 day after operation; Tf: transferrin.

### Incidence of cerebral oxygen desaturation

The incidence of cerebral oxygen desaturation was similar between the two groups [16 of 43 (37%) patients *vs*. 17 of 44 (39%) patients; *P* = 0.97]). For individual components of the composite cerebral oxygen desaturation outcome, similar incidences of SjO_2_ desaturation occurred with 70% FiO_2_ *vs*. 100% FiO_2_ [16 of 43 (37%) patients *vs*. 16 of 44 (36%) patients; *P* = 0.94], and similar incidences of rSO_2_ desaturation occurred with 70% FiO_2_ *vs*. 100% FiO_2_ [2 of 43 (5%) patients *vs*. 2 of 44 (5%) patients; *P* = 0.98; **Table 2**].

### Inflammatory biochemical indicators

Repeated-measures analysis of variance demonstrated that there was a significant interaction between time and group in the serum concentration of SOD (*P*_interaction_ = 0.005). There was a significant difference in the SOD concentration over time (*P*_time_ < 0.001), which initially decreased but then subsequently increased. The results of the Bonferroni post hoc test showed that, at Tii, the SOD concentration was significantly lower in both groups than the baseline at T_i_, and the SOD concentration was significantly lower in the 100% FiO_2_ group than in the 70% FiO_2_ group (*P* = 0.03). At T_iii_, the SOD concentration was significantly higher in both groups than at Ti and T_ii_, but there was no significant difference in the serum concentration of SOD between the two groups. We neither found significant group time interaction nor main effect for group, although there was a significant main effect for time on the serum concentrations of Tf, sFer, and IMA (*P*_time_ < 0.001; **Table 2**).

### Blood gas analysis

For the OI value, a significant group time interaction (*P*_interaction_ = 0.01) was observed, with a significant change before and after OLV (*P*_time_ < 0.001; **Table 2**). Compared with T_1_, the OI decreased significantly in both groups at T_2_, T_3_, and T_4_ and then returned to the basal level at T_5_. The OI in the 100% FiO_2_ group was significantly higher than that in the 70% FiO_2_ group at T_3_ (*P* = 0.02) and T_4_ (*P* = 0.03; **Table 2**).

The change in PA-aO_2_ in the two groups was time-dependent (*P*_time_ < 0.001) and the PA-aO_2_ in the 100% FiO_2_ group was significantly higher than that in the 70% FiO_2_ group (*P*_group_ < 0.001). However, the interaction effect showed that there was no interaction between group and time (*P*_interaction_ = 0.77; **Table 2**).

Repeated-measures analysis of variance showed a significant effect of group time interaction (*P*_interaction_ = 0.007; **Table 2**) for PaCO_2_. The PaCO_2_ was significantly higher in the 70% FiO_2_ group than in the 100% FiO_2_ group at T_2_ (*P* = 0.02), T_3_ (*P* = 0.01), and T_4_ (*P* = 0.004) (**Table 2**). There was no significant change over time in the 100% FiO_2_ group compared with the 70% FiO_2_ group.

There was a significant main effect for time in PjCO_2_ (*P*_time_ = 0.04). There was a significant difference between the two groups, with a higher PjCO_2_ in 70% FiO_2_ group compared with the 100% FiO_2_ group (*P*_group_ = 0.01). However, there was no interaction effect between group and time (*P*_interaction_ = 0.09; **Table 2**).

Although SaO_2_ was significantly different between the groups (*P*_group_ < 0.008) and changed in a time-dependent manner (*P*_time_ < 0.001), there was no significant interaction effect in SaO_2_ (*P*_interaction_ = 0.12; **Table 2**). There was neither a time effect nor an interaction effect between group and time for either SjO_2_ or Hb concentration.

## Discussion

With the extension of human life expectancy in a globally aging society, an increasing number of elderly people are receiving surgical treatment. Therefore, DNR has become one of the hotspots of clinical research in recent years.

Some studies have suggested that oxidative stress and inflammation caused by inhaling high concentrations of oxygen may damage neurons, resulting in an increased risk of DNR in patients after surgery.^18^,^36^,^37^,^38^ However, several recent clinical studies have shown that the concentration of inhaled oxygen during operations does not affect the postoperative neurocognitive function of patients. Shaefi et al.^18^ found that, in patients who underwent coronary artery bypass grafting under cardiopulmonary bypass, there was no significant difference in cognitive function between patients who received 100% inhaled oxygen and those who received 35% inhaled oxygen on the 2^nd^ day after surgery. Similarly, Lin et al.^39^ used the delirium assessment scale to evaluate the cognition of patients on the 7^th^ day after an operation and found no significant difference in the incidence of postoperative delirium between patients with 40% and 80% FiO_2_. In our study, there was no significant difference in the incidence of DNR between the 70% and 100% FiO_2_ groups one and three days after surgery.

In contrast to the view that cognitive damage is caused by hyperoxygenation, many studies have suggested that a decrease in cerebral oxygen saturation during OLV may increase the incidence of DNR. In this study, SjO_2_ values were obtained by blood gas analysis from internal jugular venous blood. The results showed no significant difference in the SjO_2_ values between the two groups at different time points, indicating that reducing the concentration of inhaled oxygen to a certain extent does not change the balance of cerebral oxygen supply and demand. In recent years, rSO_2_ has been widely used in clinics to indirectly evaluate cerebral oxygen supply and consumption for its non-invasive characteristics and regional monitoring. In this experiment, in addition to internal SjO_2_, we also applied rSO_2_ to monitor the occurrence of cerebral oxygen desaturation. The final results showed that there was no significant difference in the incidence of cerebral oxygen desaturation events between the two groups with FiO_2_ of 70% and 100%. To some extent, this result demonstrates that there is no higher risk of brain hypoxia in the 70% FiO_2_ group compared with the 100% FiO_2_ group.

Blood gas analysis results showed that there were significant differences in PA-aO_2_, OI, and SaO_2_ between the two groups, which proved that the intervention of FiO_2_ in this experiment was effective. However, the blood gas analysis results revealed no significant difference in SjO_2_ between the two groups, while there was a significant difference in SaO_2_. This indicated that a decrease in the oxygen saturation of the cerebral artery did not affect the oxygen saturation of the cerebral veins. The reason may be that when SaO_2_ decreases within a certain range, cerebral blood vessels dilate, and CBF increases compensatorily, maintaining the balance between cerebral oxygen supply and oxygen consumption. Only when the increase in CBF cannot compensate for the decrease in SaO_2_ will it cause a decrease in SjO_2_.^26^ In addition, blood gas analysis in this study also showed that PaCO_2_ and PjCO_2_ were higher in the 70% FiO_2_ group than the 100% FiO_2_ group. CO_2_ dilates brain vessels and increases CBF, which may be involved in the CBF compensation process mentioned above. It is better to observe more indicators, such as cerebral blood flow and cerebral vasospasm or dilation. Since the experimental conditions were limited, we did not observe these indicators. We hope that we can observe these indicators in future research.

Many studies have shown that in patients undergoing liver transplantation, coronary artery bypass grafting, and lumbar discectomy, there is a correlation between the occurrence of DNR and a high level of CRP in the plasma.^6^,^40^ In this study, the level of inflammatory response was evaluated by measuring the concentration of plasma hs-CRP. Compared with that 15 minutes before OLV (T_i_) and 30 minutes after OLV (T_ii_), the concentration of hs-CRP in the plasma increased significantly 1 day after surgery (T_iii_) in both groups. However, there was no significant difference in the concentration of hs-CRP in plasma between the two groups at any measurement time point. We suggested that, compared with the intervention of different FiO_2_, the inflammatory response caused by surgery is more severe, masking the difference in the inflammatory response caused by different FiO_2_ values in this study.

Previous studies have shown that oxidative stress plays a vital role in cognitive decline related to age.^41^,^42^,^43^ In patients who underwent radical resection of colon cancer, Koksal et al.^44^ found that, compared with 40% FiO_2_, the level of oxidative stress was significantly higher in patients inhaling 80% oxygen. As an antioxidant, SOD counterbalances oxidative stress.^45^ In this study, we found that the SOD concentration was significantly lower in patients receiving 100% FiO_2_ than in those receiving 70% FiO_2_ 30 minutes after OLV (T_ii_). These findings indicate that patients with high oxygen concentrations have higher oxidative stress, lower plasma SOD concentrations, and poorer antioxidant capacity.

We compared only two different FiO_2_ levels (70% and 100%) in this trial. The effect of lower fractions of inspired oxygen on DNR may be explored in the future. Cognitive tests were administered to patients only on the 1^st^ and 3^rd^ post-operative days, beginning at early discharge. Cognitive function at later time points (e.g., 6 months or 1 year) after surgery was not evaluated. It would be meaningful to investigate the effect of intraoperative FiO_2_ on long-term cognitive dysfunction.

The results of this study have substantial implications for optimizing intraoperative oxygen strategies and establishing a theoretical foundation for further scientific exploration into the optimal inspired oxygen concentration in patients undergoing OLV. Conducting high-quality, multi-center clinical studies is clinically imperative to elucidate the optimal intraoperative fractions of inspired oxygen that can effectively balance the risks of hypoxemia, oxidative stress, and inflammatory responses, ultimately reducing the incidence of DNR and improving postoperative recovery.

In conclusion, compared with 70% FiO_2_, 100% FiO_2_ did not reduce or increase the incidence of DNR on days 1 and 3 post-operatively; 100% FiO_2_ improved patients’ oxygenation during OLV but did not decrease the incidence of cerebral oxygen desaturation; and 100% FiO_2_ aggravated oxidative stress but did not cause an excessive inflammatory response in patients with OLV.

## Data Availability

*Data will be made available on request*.
